# “Discharge from Gastroenterology, Readmission to Psychiatry”: Function and Management of Recurrent Foreign-Body Ingestion in a Patient with Borderline Personality Disorder

**DOI:** 10.1192/j.eurpsy.2026.11915

**Published:** 2026-07-31

**Authors:** M. Sobredo Vega, P. Nava Garcia, M. E. Arévalo Gil, S. P. Orrego Molina, M. R. López Urbán, M. J. Sánchez Artero, F. Pascual Pinazo

**Affiliations:** 1 Hospital Universitario Infanta Leonor; 2 Hospital General Universitario Gregorio Marañón, Madrid, Spain

## Abstract

**Introduction:**

Deliberate foreign-body ingestion (DFBI) is an under-reported form of self-injury, frequently associated with borderline personality disorder (BPD). It represents a complex clinical and ethical challenge for mental-health and medical teams, particularly when episodes occur within hospital settings.

**Objectives:**

To describe the clinical features, functions, and interdisciplinary management of repetitive DFBI in a patient with BPD, and to reflect on its psychodynamic meaning and institutional impact.

**Methods:**

Clinical data were collected from repeated emergency and inpatient admissions. Psychiatric evaluation and psychodynamic formulation were integrated with reports from gastroenterology, nursing, and endoscopy services. Regular multidisciplinary case conferences and team supervision were implemented to enhance coordination and reflective functioning.

**Results:**

The patient, a 46-year-old woman diagnosed with BPD, presented over forty episodes of DFBI involving razor blades, batteries, keys, and metallic clips, most of them occurring during emergency or short-stay psychiatric admissions. Radiological and endoscopic examinations repeatedly confirmed the presence of sharp objects without major complications so far, though cumulative gastrointestinal risk was significant. Psychodynamically, the behaviour served multiple functions: affect regulation, embodiment of aggression, control over a perceived intrusive environment, and elicitation of care. These behaviours elicited strong counter-transference reactions among staff, including frustration, fear, and feelings of manipulation, which were addressed through team discussions and supervision. Pharmacological support and integrative psychotherapeutic strategies focusing on mentalization and emotional regulation achieved partial and time-limited efficacy. Given the chronicity and persistence of DFBI, the patient was eventually admitted involuntarily to a long-stay psychiatric rehabilitation unit for ongoing containment and treatment.

**Conclusions:**

Repetitive DFBI in BPD should be conceptualized as a maladaptive affect-regulation strategy rather than a primarily suicidal act. Effective management requires consistent institutional policies, clear boundaries, and coordinated interdisciplinary planning. Regular staff supervision is essential to prevent reactive or punitive responses and to maintain a coherent therapeutic frame across medical and psychiatric services.

**Disclosure of Interest:**

None Declared

